# Quercetin and Naringenin Provide Functional and Antioxidant Protection to Stored Boar Semen

**DOI:** 10.3390/ani10101930

**Published:** 2020-10-21

**Authors:** Eva Tvrdá, Mégane Debacker, Michal Ďuračka, Ján Kováč, Ondřej Bučko

**Affiliations:** 1 Department of Animal Physiology, Faculty of Biotechnology and Food Sciences, Slovak University of Agriculture, Tr. A. Hlinku 2, 949 76 Nitra, Slovakia; michaelduracka@gmail.com (M.Ď.); jan.johnny.kovac@gmail.com (J.K.); 2 Condorcet—Hainaut Provincial High School, Chemin du Champ de Mars 17, 7000 Mons, Belgium; megane.debacker@hotmail.com; 3 Department of Animal Husbandry, Faculty of Agrobiology and Food Resources, Slovak University of Agriculture, Tr. A. Hlinku 2, 949 76 Nitra, Slovakia; ondrej.bucko@uniag.sk

**Keywords:** quercetin, naringenin, boars, spermatozoa, semen extender, antioxidants

## Abstract

**Simple Summary:**

A significant effort has been devoted to the enhancement of boar sperm preservation media and techniques, which have become a critical pillar of modern swine production. Despite the availability of numerous semen extenders, a higher antioxidant protection of male gametes is highly required, which may be achieved by the supplementation of natural biomolecules such as quercetin and naringenin. In this regard, we have performed a number of experiments in order to define the optimal concentration range of both biomolecules that could ensure a higher structural integrity, functional activity, and antioxidant profile of boar spermatozoa subjected to short-term storage. The beneficial outcomes achieved in this study shall be tested in vivo at our collaborating pig farm, with their potential contribution to the optimization of the use of stored boar semen in the porcine breeding industry.

**Abstract:**

In this study, we evaluated the impact of 5–50 μM quercetin (QUE) and naringenin (NAR) on extended boar spermatozoa in the BTS (Beltsville Thawing Solution) medium for 72 h. Spermatozoa motion, membrane, acrosome, and DNA integrity were investigated immediately after sample dilution (0 h) as well as after 24 h, 48 h, and 72 h of semen storage. Furthermore, reactive oxygen species (ROS) and superoxide production, as well as the extent of oxidative damage to the sperm proteins and lipids, were assessed to determine the potential of QUE and NAR to prevent a potential loss of sperm vitality due to oxidative stress development. Our results indicate that the most notable parameter influenced by QUE was the mitochondrial activity, which remained significantly higher throughout the experiment (*p* < 0.001 and *p* < 0.0001; 10 μM), and which correlated with the most prominent maintenance of sperm motility (*p* < 0.01, 48 h; *p* < 0.05, 72 h). A significant membrane stabilization (*p* < 0.01, 24 h and 48 h; *p* < 0.0001, 72 h) and prevention of lipid peroxidation (*p* < 0.05, 24 h and 48 h; *p* < 0.01, 72 h) was primarily observed following administration of 10 and 25 μM NAR; respectively. Administration of 10 μM QUE led to a significant decrease of superoxide (*p* < 0.0001, 48 h and 72 h) while the most notable decline of ROS generation was recorded in the case of 10 and 25 μM NAR (*p* < 0.001). This study may provide new information on the specific mechanisms of action involved in the favorable effects of natural biomolecules on spermatozoa.

## 1. Introduction

Over the past decades, a substantial effort has been invested in the evolution of modern reproductive technologies that are nowadays an indispensable part of the global swine industry. Among these, artificial insemination (AI) has become the technique of choice for most countries with an intensive pig production, as more than 90% of the sows in Western Europe are currently being bred artificially [1]. As opposed to natural mating, AI has become an efficient and time-saving tool to introduce superior genes into sow herds while carrying a minimal risk of disease [2]. Nevertheless, the outcome of AI by and large depends on the semen quality.

Previous research has shown that semen extenders enable the male gametes to maintain their activity and fertilization ability for a substantially longer time period before insemination. As such, the use of preservation media designed particularly for boar semen has become routine since the 1970s [3]. Although diverse commercially available extenders are to be found in the industry, semen diluents can be generally divided into extenders designed for short-term preservation (1–3 days), and extenders for long-term semen preservation (over 4 days) [4]. Among short-term semen diluents commonly used for the production of insemination doses at the farm itself, BTS (Beltsville Thawing Solution) has become one of the most popular options [3,4].

Despite a relatively high preservation capacity of modern semen diluents, a substantial part of spermatozoa may be lost due to oxidative stress that has been notoriously associated with semen processing and storage [5,6]. Spermatozoa are well known for having a limited capacity to counteract the deleterious consequences of reactive oxygen species (ROS) overproduction. Excessive damage to sperm lipids, proteins, and DNA as a result of oxidative insults may impair the functional activity and fertilization potential of male gametes [7]. Boar spermatozoa are often affected by the occurrence of proximal or distal cytoplasmic droplets, which have been established as prime sources of intracellular ROS in semen and associated with severe oxidative alterations to the sperm quality [8]. Furthermore, a relatively high number of leukocytes and macrophages as an additional source of ROS is often to be found in boar semen [9]. As such, a common research goal is to further enhance the efficiency of boar semen extenders, particularly by their enrichment with antioxidant supplements.

The use of biomolecules of plant origin for the improvement of male reproductive performance has become a modern trend in recent years. Flavonoids, a widely distributed family of phytochemicals, possess numerous beneficial effects on animal health and production [10]. Numerous natural flavonoids have been studied in relation to male reproductive cells and tissues, among which quercetin (QUE) and naringenin (NAR) merit further attention. QUE (3,30,40,5,7-pentahydroxylflavone) is a flavonol-type flavonoid present in citrus fruits, berries, herbs and spices, tea, cocoa, as well as red wine, and fruit juices [11]. NAR (2,3-dihydro-5,7-dihydroxy-2-(4-hydroxyphenyl)-4H-1-benzopyran-4-one) is a natural flavonoid belonging to flavanones, commonly available in tomatoes, bergamot, and citrus fruits [12]. Both biomolecules have been detected in ethnopharmacological products and reported to exhibit anticarcinogenic, anti-inflammatory, antibacterial, antiviral, and cardioprotective properties. Moreover, both possess a significant antioxidant activity [11,12].

Preliminary reports suggest that both flavonoids have the ability to protect the sperm plasma membrane and acrosome structures, mitochondrial function, and DNA stability in rabbit [13] and bovine [14] spermatozoa subjected to induced oxidative stress. Their potential to provide protection to the structure and function of cryopreserved goat [15], ram [16], bull [17], rooster [18] or stallion spermatozoa [19] subjected to cryopreservation has been indicated as well. Nevertheless, both biomolecules have received relatively little attention with respect to porcine semen. In fact, only two recently published reports suggest an improvement in the vitality of processed boar spermatozoa following exposure to QUE or NAR [20,21]. As such, the aim of this study was to evaluate the in vitro effect of quercetin and naringenin on selected structural, functional, and oxidative parameters of extended boar semen subjected to short-term liquid storage.

## 2. Materials and Methods 

### 2.1. Semen Collection and Dilution 

Two hours before collection, the BTS medium (Minitüb, Tiefenbach, Germany) was prepared as per the instructions of the manufacturer and supplemented with different concentrations (5, 10, 25, 50 µM final concentration) either of QUE (Sigma-Aldrich, St. Louis, MO, USA) or NAR (Sigma-Aldrich, St. Louis, MO, USA) dissolved in 0.5% DMSO (Sigma-Aldrich, St. Louis, MO, USA). The control was enriched with 0.5% DMSO.

Ejaculates were collected from 7 adult (2–3 years old) Duroc boars housed at the pig farm Terezov (Hlohovec, Slovakia). A total of 28 semen samples (4 per boar) were obtained during the period of three months (March–May 2019). The sperm rich fraction was obtained by a qualified technician using the gloved-hand technique, and raw semen was transported to the Andrology Laboratory at the AgroBioTech Research Centre, Slovak University of Agriculture in Nitra, Slovakia in an isothermal vessel (37 °C) within 30 min following collection. 

The animals and sample collection were carefully handled in accordance with ethical guidelines as stated in the Slovak Animal Protection Regulation RD 377/12, which conforms to European Union Regulation 2010/63. Since semen collection is routinely performed at the farm Terezov, causing no harm or discomfort, a special Ethical Approval was not needed for this type of experiments.

Each sample was subjected to a primary assessment of semen volume, as well as sperm concentration and motility. All samples accomplished the given criteria (volume > 200 mL, concentration > 200 × 10^6^ sperm/mL, motility > 70%, leukocytospermia-free) for subsequent experiments.

Each ejaculate was divided into 9 equal fractions and each part was diluted either with the control extender or with one of the 8 experimental media at 30 °C using a dilution ratio of 1:20. The diluted samples were stored under controlled temperature conditions (16–18 °C). Specific semen assessments were performed immediately following dilution (0 h) as well as 24 h, 48 h, and 72 h post-dilution.

Prior to each round of analysis, 10 mL of diluted semen was pre-warmed in a water bath to 37 °C and subjected to the evaluation of the sperm motion behavior, stability of the plasma membrane and acrosome, DNA integrity, mitochondrial activity, ROS, and superoxide production. Furthermore, each sample was centrifuged (800× *g*, 10 min), the resulting sperm fraction was treated with RIPA buffer (Sigma-Aldrich, St. Louis, MO, USA) containing protease inhibitor (Sigma-Aldrich, St. Louis, MO, USA) and sonicated at 28 kHz for 30 s. The mixture was centrifuged (11,828× *g*, 4 °C, 10 min), the obtained lysates were purified and stored at −80 °C for the assessment of protein and lipid oxidation.

### 2.2. Sperm Motility

Sperm motility (MOT; %) was quantified using the HTM TOX IVOS II. Computer-assisted sperm analysis (CASA) system (Hamilton-Thorne Biosciences, Beverly, MA, USA). The analysis setup was adjusted to the following cut-off values: number of frames: 45; frame rate: 60 Hz; min contrast: 46; min cell size: 7 pixels; min contrast: 30; cell size: 7 pixels; cell intensity: 50; static head size: 0.80–4.93; static head intensity: 0.49–1.68; static elongation: 22–84. The Makler counting chamber (Sefi Medical Instruments, Haifa, Israel) served to load the sample into the CASA system, and a minimum of 1000 spermatozoa were evaluated.

### 2.3. Sperm Mitochondrial Activity

The sperm mitochondrial function was assessed with the colorimetric MTT test. Briefly, MTT tetrazolium (Sigma-Aldrich, St. Louis, MO, USA) was dissolved in Dulbecco’s PBS (phosphate-buffered saline without Ca^2+^ or Mg^2+^; Sigma-Aldrich, St. Louis, MO, USA) at a concentration of 5 mg/mL. Ten μL of the staining solution was added to 200 μL of each sample. Finally, 80 μL acidified isopropanol (Centralchem, Bratislava, Slovakia) were administered to stop the reaction.

The amount of formazan formed as a result of the mitochondrial functionality was determined at a wavelength of 570 nm against 620 nm as reference using a microplate spectrophotometer (Promega, Madison, WI, USA). The mitochondrial activity of the experimental groups is expressed as a percentage of the control, set to 100% [20].

### 2.4. Membrane Integrity

For the membrane integrity analysis, 1 × 10^6^ spermatozoa were adjusted to 100 μL with PBS, stained with 10 μL CFDA (carboxyfluorescein diacetate; Sigma-Aldrich, St. Louis, MO, USA; 0.75 mg/mL in DMSO) and 10 μL DAPI (4′,6-diamidino-2-phenylindole; Sigma-Aldrich, St. Louis, MO, USA; 1 μM in PBS). Following incubation (37 °C, dark conditions, 15 min), the samples were centrifuged for 5 min at 300× *g*, and the pellet was washed with 100 µL PBS. Following a second round of centrifugation and washing, the cells were resuspended in 100 µL PBS, transferred to a dark 96-well plate and assessed with the Glomax Multi+ combined spectro-fluoro-luminometer (Promega, Madison, WI, USA). The UV filter (EX 365, EM 410–460) was used in case of DAPI, while the blue filter (EX 490, EM 510–570) served to assess the CFDA signal. Cells exhibiting CFDA-positivity were considered to be membrane-intact (%).

### 2.5. Acrosome Integrity

In case of the acrosome integrity, 1 × 10^6^ cells were diluted to 100 μL using PBS, followed by the administration of 100 μL PNA (peanut agglutinin, FITC conjugate; Sigma-Aldrich, St. Louis, MO, USA; 10 μM in PBS) and 10 μL DAPI. Following incubation (37 °C, 30 min), the samples were transferred to a dark 96-well plate and analyzed using the Glomax Multi+ combined spectro-fluoro-luminometer (Promega, Madison, WI, USA) using appropriate filter settings (UV filter in the case of DAPI, green filter with respect to PNA-FITC). Spermatozoa exhibiting PNA-negativity were defined as acrosome-intact (%).

### 2.6. DNA Fragmentation

Sperm DNA damage was assessed using the Halomax commercial kit (Halotech DNA, Madrid, Spain). Briefly, 20 μL of the sample were mixed with low-melting point agarose. Ten μL of the mixture were transferred onto glass slides pre-coated with agarose, covered with coverslips, and placed at 4 °C for 5 min. Subsequently, the samples were subjected to a lysis solution (5 min), distilled water (5 min), 70% and 100% ethanol (2 min each) and finally air-dried [22].

The slides were dyed with SYBR Green (2 μg/mL) (Sigma-Aldrich, St. Louis, MO, USA) in Vectashield (Vector Laboratories, Burlingame, CA, USA) and at least 300 cells per slide were scored under an epifluorescence microscope with a ×40 magnification objective (Leica Microsystems, Wetzlar, Germany). The proportion of spermatozoa with fragmented DNA is expressed as a percentage.

### 2.7. Reactive Oxygen Species (ROS) Generation

The extent of ROS formation was quantified using chemiluminescence taking advantage of luminol (Sigma-Aldrich, St. Louis, MO, USA). The tested specimens contained 10 μL 5 mM luminol and 400 μL sample. Negative controls contained 400 μL of each control or experimental extender. Positive controls consisted of 400 μL of each extender and 50 μL hydrogen peroxide (H_2_O_2_; 30%; 8.8 M; Sigma-Aldrich, St. Louis, MO, USA). The chemiluminescent reaction was measured in fifteen 1-min cycles on 48-well microplates using the Glomax Multi+ combined spectro-fluoro-luminometer (Promega, Madison, WI, USA). The extent of ROS production by spermatozoa is expressed in relative light units (RLU)/s/10^6^ sperm [23].

### 2.8. Superoxide Production

The nitroblue-tetrazolium (NBT) test was selected to evaluate the superoxide generation within spermatozoa. The assay employs nitroblue tetrazolium chloride, which reacts with the superoxide radical, leading to the generation of intracellular blue NBT formazan deposits [24].

The NBT salt (Sigma-Aldrich, St. Louis, MO, USA) was dissolved in Dulbecco’s PBS containing 1.5% DMSO and added to 100 μL of each cell suspension. Following incubation (1 h; 37 °C), the cells were washed twice with PBS and centrifuged at 300× *g* for 10 min. Finally, the cell suspension was subjected to 2 M potassium hydroxide (KOH; Centralchem, Bratislava, Slovakia) dissolved in DMSO. The amount of superoxide was assessed spectrophotometrically at a wavelength of 620 nm against 570 nm using a microplate spectrophotometer (Promega, Madison, WI, USA). Data obtained from the experimental groups are expressed in percentage of the control, which was set to 100% [20].

### 2.9. Protein Oxidation

Oxidative damage to proteins was assessed using the 2,4-dinitrophenylhydrazine (DNPH) method, suitable for the quantification of protein carbonyls [25]. One mL of the sperm lysate was subjected to trichloroacetic acid (TCA; 20% *w*/*v*; Sigma-Aldrich, St. Louis, MO, USA) treatment, subsequently mixed with 1 mL DNPH (10 mM in 2 N HCl; Sigma-Aldrich, St. Louis, MO, USA) and incubated at 37 °C for 1 h. The mixture was treated again with 1 mL TCA, incubated at 4 °C for 10 min and centrifuged (11,828× *g*, 10 min). The pellet was washed three times with 1 mL ethanol/ethyl acetate (1/1; *v*/*v*; Sigma-Aldrich, St. Louis, MO, USA) and finally resuspended in 1 mL 6 M guanidine HCl (Sigma-Aldrich, St. Louis, MO, USA). The absorbance was measured at 360 nm with the Cary 60 spectrophotometer (Agilent Technologies, Santa Clara, CA, USA). Guanidine HCl (6M) was used as a blank. The molar absorption coefficient of 22,000/M/cm was applied for the calculation of the protein carbonyl concentration in the samples. The extent of protein oxidation is expressed in nmol protein carbonyls/mg protein [23].

### 2.10. Lipid Peroxidation

Malondialdehyde (MDA) concentration as the prime marker of lipid peroxidation (LPO) was evaluated with the TBARS (thiobarbituric acid reactive substances) assay, modified for a microplate. Each sample was subjected to treatment with 5% sodium dodecyl sulphate (Sigma-Aldrich, St. Louis, MO, USA), and subsequently boiled (90–100 °C) in the presence of 0.53% thiobarbituric acid (TBA; Sigma-Aldrich, St. Louis, MO, USA) dissolved in 20% acetic acid (pH 3.5; Centralchem, Bratislava, Slovakia) for 1 h. Afterwards, the samples were cooled down on ice for 10 min and centrifuged (1750× *g*, 10 min). The supernatant was used to quantify MDA at 540 nm with the help of a microplate spectrophotometer (Promega, Madison, WI, USA). MDA concentration is expressed as μmol/g protein [23].

### 2.11. Data Normalization

To normalize the data collected from the DNPH and TBARS assays, the protein concentration in each lysate was quantified with the DiaSys Total Protein (DiaSys, Holzheim, Germany) commercial kit and the RX Monza analyzer (Randox, Crumlin, UK). The protocol followed the Biuret method and the colorimetric reaction was evaluated at 540 nm. 

### 2.12. Statistical Analysis

The collected data were statistically evaluated with the GraphPad Prism program (version 8.4.3 for Mac; GraphPad Software, La Jolla, CA, USA). Descriptive statistical characteristics (mean, standard deviation) together with One-way ANOVA and Dunnett’s post-test were selected for the analysis. The level of significance was set at **** *p* < 0.0001; *** *p* < 0.001; ** *p* < 0.01; * *p* < 0.05.

## 3. Results

### 3.1. The Effect of Quercetin and Naringenin on the Sperm Functional Activity

The effects of selected flavonoids on the sperm motion behavior are detailed in Table 1. At Time 0 h, MOT did not differ significantly when the Control was compared with the experimental groups. The assessment at Time 24 h revealed that the decline of sperm MOT was significantly slower in the samples supplemented with 5–25 µM QUE (*p* < 0.001 in case of 5 µM QUE; *p* < 0.0001 with respect to 10 µM QUE; *p* < 0.01 in relation to 25 µM QUE) as well as with 10–50 µM NAR (*p* < 0.001 with respect to 10 µM NAR; *p* < 0.05 in case of 25 and 50 µM NAR). Following 48 h, a significantly higher MOT was detected in case of 5 and 10 µM QUE (*p* < 0.01) as well as 10 µM (*p* < 0.01) and 25 µM NAR (*p* < 0.05) in comparison to the Control. Interestingly, a significant (*p* < 0.05) MOT inhibiting effect was observed in case of the experimental group enriched with 50 µM QUE. The final assessment (72 h) revealed that 10 µM QUE and 25 µM NAR had the most significant MOT-preserving effect (*p* < 0.05) in comparison with the Control. On the other hand, a significant MOT-suppressing impact was recorded in case of 25 µM (*p* < 0.05) and 50 µM QUE (*p* < 0.0001) (Table 1).

**Figure 1:**
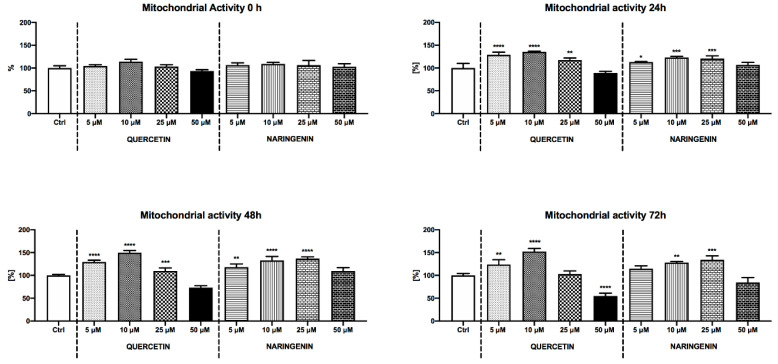
Mitochondrial activity of boar spermatozoa extended in the presence of quercetin or naringenin. Each bar represents mean (± S.D.) optical density as the percentage of the control, which was set to 100%, and the data are expressed as a% of the control value. The data were obtained from four independent experiments. Mean ± S.D. * *p* < 0.05; ** *p* < 0.01; *** *p* < 0.001; **** *p* < 0.0001.

The MTT assay revealed the first differences in the mitochondrial activity after 24 h in case of boar spermatozoa exposed to 5–25 µM QUE and NAR, reflected in a significantly higher vitality (*p* < 0.05 in case of 5 µM NAR; *p* < 0.01 in terms of 25 µM QUE; *p* < 0.001 with respect to 10 and 25 µM NAR; *p* < 0.0001 in relation to 5 and 10 µM QUE) when compared to the control (Figure 1). Beneficial effects of 5–25 µM QUE and NAR on the mitochondrial metabolism were observed following 48 h as well. At the end of the experiment, the highest mitochondrial activity was detected in the experimental group subjected to 5 and 10 µM QUE (*p* < 0.01 with respect to 5 µM and *p* < 0.0001 in case of 10 µM QUE). A significantly higher mitochondrial activity was also detected in case of 10 µM (*p* < 0.01) and 25 µM NAR (*p* < 0.001) when compared to the control. Inversely, 50 µM QUE exhibited significant time-dependent toxic effects on the mitochondrial activity at 72 h in comparison to the control (*p* < 0.0001).

### 3.2. The Effect of Quercetin and Naringenin on the Sperm Structural Integrity

Assessment of spermatozoa with intact membranes was carried out using the fluorescently labelled CFDA probe. A significant protective effect of 10 µM QUE (*p* < 0.01) as well as 10 µM (*p* < 0.01) and 25 µM NAR (*p* < 0.05) on the sperm plasma membrane became evident after 24 h and was maintained even following 48h of sperm storage (Table 2). Interestingly, a significantly higher proportion of spermatozoa with damaged membranes was observed following a 48 h exposure to 50 µM QUE when compared to the control (*p* < 0.01). The final assessment (72 h) revealed a significantly higher number of spermatozoa without membrane damage in the experimental groups exposed to 5 µM (*p* < 0.01) and 10 µM QUE (*p* < 0.001) as well as to 5 µM (*p* < 0.001), 10 µM and 25 µM NAR (*p* < 0.0001) in comparison with the control. The highest proportion of spermatozoa exhibiting damage to the plasma membrane was recorded in case of the experimental group supplemented with 50 µM QUE, which was significantly higher in comparison with the control (*p* < 0.01).

Similarly to the membrane stability, the PNA-based fluorescent analysis revealed beneficial effects of 10 µM QUE as well as 10 µM and 25 µM NAR on the stability of the sperm acrosome, which became significant (*p* < 0.05 in case of 10 µM QUE and *p* < 0.01 with respect to 10 µM and 25 µM NAR) following 48 h of semen storage (Table 3). These protective properties became more pronounced and significant towards the end of the experiments (*p* < 0.01 in terms of 10 µM QUE and 25 µM NAR; *p* < 0.001 with respect to 10 µM NAR; Time 72 h) when compared to the control. The lowest proportion of boar spermatozoa with intact acrosomal structures was detected in the experimental group subjected to 50 µM QUE, which was significantly decreased in comparison with the control (*p* < 0.001), particularly at Times 48 h and 72 h (Table 3).

As a variety of studies point out the importance of preservation of the sperm DNA integrity to achieve a successful AI cycle followed by a confirmed pregnancy, we chose to study the impact of QUE and NAR on the sperm DNA stability using the chromatin dispersion test. Our results revealed DNA-stabilizing effects, particularly in the case of 10 μM QUE as well as 10 and 25 μM NAR, which became evident after 24 h (*p* < 0.05) and remained significant throughout the entire experiment (*p* < 0.05). On the other hand, the highest QUE concentration exhibited genotoxic properties as a significantly higher incidence of fragmented sperm DNA was observed in this case (*p* < 0.05) when compared to the control following 48 and 72 h of storage (Table 4).

### 3.3. The Effect of Quercetin and Naringenin on the Oxidative Profile of Boar Spermatozoa

**Figure 2:**
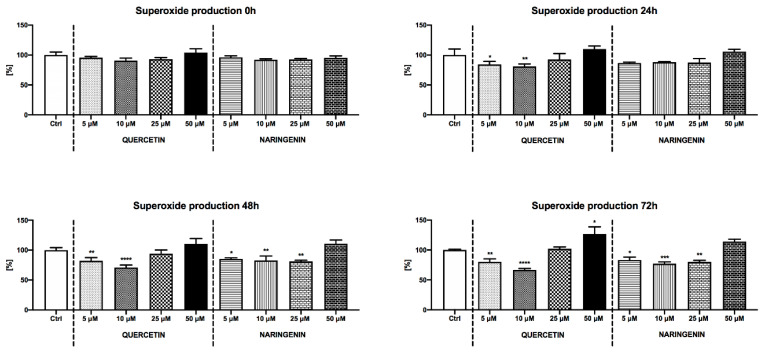
Intracellular superoxide production of boar spermatozoa extended in the presence of quercetin or naringenin. Each bar represents mean (± S.D.) optical density as the percentage of the control, which was set to 100% and the data are expressed as a% of the control value. The data were obtained from four independent experiments. Mean ± S.D. * *p* < 0.05; ** *p* < 0.01; *** *p* < 0.001; **** *p* < 0.0001.

To confirm the potential antioxidant properties of QUE and NAR, we used a luminometric approach using luminol as the probe, which has been extensively used to study the global ROS production by sperm in mammals (Table 5) [14,23]. Furthermore, we focused on the production of superoxide which is considered to be the principal free radical generated by respiring cells (Figure 2). At 24 h the amount of ROS significantly decreased following exposure to 10 μM QUE as well as 5–25 μM NAR in comparison with the control (*p* < 0.05). This ability to prevent ROS overproduction remained significant following 48 h (*p* < 0.05 in case 5 μM NAR; *p* < 0.01 with respect to 10 μM QUE and 10 μM NAR; *p* < 0.001 in relation to 25 μM NAR) and 72 h (*p* < 0.001 in terms of 10 μM QUE and 5 μM NAR; *p* < 0.0001 with respect to 10 and 25 μM NAR). On the other hand, the superoxide production at 24 h was significantly reduced in the experimental groups supplemented with 5 μM (*p* < 0.05) and 10 μM QUE (*p* < 0.01) in comparison with the control. Significant superoxide quenching properties of both QUE concentrations (*p* < 0.01 for 5 μM and *p* < 0.0001 in case of 10 μM) were detected following 48 h as well as 72 h. Furthermore, a significantly decreased amount of superoxide was recorded in the experimental groups enriched with 5 μM (*p* < 0.05), 10 μM and 25 μM NAR (*p* < 0.01 and *p* < 0.001; respectively) following 48 h and 72 h of storage.

The DNPH assay revealed that particularly QUE exhibited significant protective effects on the sperm proteins against possible oxidation (Table 6). A significantly lower concentration of protein carbonyls was detected at 72 h in the experimental groups supplemented with 5 μM (*p* < 0.05) and 10 μM QUE (*p* < 0.001) in comparison with the control. Furthermore, a significant decline of oxidized proteins was observed in case of 25 μM NAR when compared to the control following 72 h of storage.

In case of LPO, NAR exhibited a stronger potential to cease the peroxidative damage to the sperm lipids (Table 7). A significantly decreased MDA concentration was detected following a 24 h—exposure to 25 μM NAR (*p* < 0.05) in comparison with the control. After 48 h, a significantly lower LPO was observed in the case of samples subjected to the treatment with 10 and 25 μM NAR as well as 10 μM QUE (*p* < 0.05). This significant lipoprotective effect was observed during the final (Time 72 h) assessment as well (*p* < 0.05 in case of 10 μM QUE; *p* < 0.01 with respect to 10 and 25 μM NAR).

## 4. Discussion

Based on a convincing body of evidence indicating favorable effects of natural biomolecules with antioxidant properties on the quality of stored semen, this study aimed to elucidate the impact of quercetin or naringenin on (1) the sperm stability and activity, as well as (2) selected parameters reflecting the oxidative profile of boar spermatozoa subjected to short-term storage.

The collected data indicate that the presence of specific concentrations of both biomolecules in the BTS extender results in a significant improvement of the sperm structural integrity and functional activity as well as a significant stabilization of the oxidative profile of the male gametes following storage. Nevertheless, each biomolecule was revealed to operate through a different mechanism of action within the reproductive cell.

It has become a global fact that oxidative insults associated with sperm storage over an extended period of time may lead to the disruption of structures critical for the survival of male reproductive cells [6]. Numerous reports have claimed that boar spermatozoa subjected to in vitro storage may suffer from morphological alterations to the head and/or tail, which are associated with alterations to the plasma membrane. At the same time, exposure of male gametes in the ex vivo environment and elevated oxygen tension may result in mitochondrial dysfunction, loss of ATP synthesis, and motility inhibition [26,27,28]. In the meantime, it must be remembered that maintenance of satisfactory sperm motility is a prime prerequisite for a successful AI and subsequent pregnancy in swine breeding programs [27].

Our CASA analysis revealed that a concentration range of 5–10 μM QUE and 10–25 μM NAR ensured the best sperm motility preservation over a 3-day period of storage, which is the maximum time recommended for the BTS medium [3]. In the case of QUE, our observations are in agreement with previous reports on goat [15], human [29], and ram spermatozoa [16], suggesting that exposure of male gametes to particularly lower QUE concentrations may lead to a higher sperm motion behavior. Furthermore, pivotal experiments on cryopreserved boar spermatozoa [21] suggest that low QUE concentrations administered to the freezing and thawing medium improve sperm motility and subsequent fertilization potential. The most likely explanation for these observations has been linked to the unique chemical structure of QUE, enabling its ability to either directly inhibit ROS overgeneration or to avoid ROS leakage as a result of mitochondrial dysfunction. On the other hand, it must be remembered that QUE is well-known for its dichotomy, as high concentrations of this biomolecule have either no or negative effects on the sperm motion characteristics [30,31]. As suggested by Khanduja et al. [30] and Córdoba et al. [32], this inhibition may be most likely caused by the blockage of the microtubules located in the sperm flagellum, since QUE is known to promote calcium accumulation by decreasing the activity of Ca^2+^-dependent ATPase.

In the case of naringenin, exposure to 10 and 25 μM NAR ensured a highly satisfactory motility of boar spermatozoa, particularly after 24 and 48 h. These data are in agreement with previous in vitro reports on chilled or frozen rooster and boar semen [20,21], suggesting that NAR may have beneficial effects on the motility behavior as a result of its lipophilic nature and antioxidant protection of the membranous structures crucial for a proper internal milieu of male gametes. Inversely, our results indicate that concentrations above 50 μM NAR may have deteriorating effects on the maintenance of sperm motion, as previously shown by Moretti et al. [31] on human spermatozoa.

The MTT assessment revealed that in vitro exposure of spermatozoa to both flavonoids leads to a significant protection of the mitochondrial function, mirroring the preservation of sperm motility. The logical explanation for such observation would be that axonemes and dense fibers located the central part of spermatozoa are covered by mitochondria, responsible for the production of ATP, directly involved in the sperm motion [33]. Phenolic compounds have been recognized to exhibit protective effects on the mitochondrial structure and function, with QUE itself being revealed as a strong mitochondrial protector on numerous occasions. The most prominent oxidative stress-preventing characteristic of QUE is tied to its ability to directly inhibit ROS formation by enzymatic or non-enzymatic systems, particularly NADPH oxidase and NADH-dependent oxidoreductase, localized directly in the sperm mitochondria [33,34]. On the other hand, arguable dose-dependent effects of QUE towards the sperm mitochondria were observed by Silva et al. [15]. In a comparative analysis between two potent polyphenols (resveratrol and quercetin), QUE exhibited a higher toxicity towards sperm motility and viability than resveratrol administered at the same dose, although the antioxidant potential of QUE was more pronounced.

In the case of NAR, it may be hypothesized that the molecule exhibits its mitochondrial-protecting effects through a different mechanism of action. As revealed by Mehdipour et al. [18], NAR supplementation to frozen rooster spermatozoa led to significant reduction of mitochondrial ruptures and a subsequent activation of the mitochondrial-mediated apoptotic pathway. A more favorable ratio of pro-apoptotic Bax/anti-apoptotic Bcl-2 genes was observed following exposure to NAR, which lead to a decreased release of cytochrome C and a subsequent deactivation of caspase-3 [35]. As such, we may speculate that while QUE offers protection to the mitochondria by a direct prevention of mitochondrial ROS overproduction, NAR interferes with the apoptotic machinery mediated by an eventual mitochondrial dysfunction.

The fluorescent assessment of the membrane and acrosome integrity revealed significant protective effects of NAR on the membranous structures supporting a proper intracellular balance and a successful acrosome reaction of extended boar spermatozoa. Membrane- and acrosome-protecting properties of NAR have been already reported by Mehdipour et al. [18]. Inversely, a relatively small effect of NAR against membrane and acrosomal ruptures, as well as a premature acrosome reaction, was observed by Moretti et al. [31]. This discrepancy may be explained by their experimental setting, in which human spermatozoa were exposed to a high prooxidant environment, which might have caused damage to male gametes beyond repair. It has been previously shown that NAR diffuses into the membranes and is able to interfere particularly with H_2_O_2_ [36]. Furthermore, NAR has been reported to enhance the activity of specific detoxifying enzymes and to affect the fluidity of the membrane, modulating its permeability to oxygen and oxygen-derived molecules [37].

In the case of QUE, our data do not correlate with those obtained by Silva et al. [15] studying goat semen, although QUE administration to human [31] rabbit [13] or bull semen [14] has been shown to be associated with a lower degree of damage to the membranous structures of male gametes. Nevertheless, it must be noted that the majority of the aforementioned studies focused on male gametes following an induction of a stressful situation (induced oxidative stress, cryopreservation, etc.), which may lead to the assumption that the protective effect of natural antioxidants may become evident only in situations of extreme stress, which may not necessarily correspond to a physiological situation, under which high QUE doses may exhibit toxic effects on the structural integrity of the plasma membrane of male gametes.

Our results reveal that certain doses of both biomolecules were able to prevent excessive DNA fragmentation of stored boar spermatozoa, although such beneficial effects were observed in the case of a wider concentration range of NAR. It has been previously postulated that DNA-protective effects of flavonoids may be mediated through genomic pathways as evidenced by in vivo studies, but also by non-genomic mechanisms. As discussed before, NAR acts as a very efficient H_2_O_2_ scavenger. This feature could also be responsible for its DNA-protective properties, an assumption complementing Oršolić et al. [38]. If NAR is able to neutralize excessive H_2_O_2_ in the sperm membranes, hydrogen peroxide will not be able to undergo further intracellular conversion to the hydroxyl radical, and thus DNA damage can be prevented. As hypothesized by Adana et al. [39] NAR could also affect enzymes responsible for the repair of sperm DNA strand breaks, or to upregulate the antioxidant pathway to subsequently neutralize excessive ROS. A direct beneficial effect of NAR on genes responsible for DNA-associated apoptotic changes was furthermore detected by Mehdipour et al. [18], which was well-reflected in a higher fertilization potential of frozen-thawed rooster spermatozoa.

A direct ROS-quenching effect has been associated with the DNA-protective effects of QUE. As suggested previously, protective effects of QUE on the mitochondria and its straight-forward antioxidant abilities could decrease a possible ROS leakage from the sperm mitochondria to the nucleus, minimizing the exposure of the sperm chromatin to oxidative insults. Correspondingly to our findings, Avdatek et al. [17], as well as Gibb et al. [19], found that QUE significantly reduced DNA fragmentation in cryopreserved bovine and stallion spermatozoa. Another study suggested that QUE minimized the chromatin distortion caused by the exposure of human spermatozoa to tert-butylhydroperoxide (TBHP) and could be considered as a supplement for human sperm processing process [31]. Inversely, Seifi-Jamadi et al. [40] observed that the addition of QUE did not exhibit any beneficial effects on the percentage of extended Turkmen stallion spermatozoa with an intact DNA molecule, which may be explained by relatively high QUE concentrations used (100–300 μM) in comparison to our experimental settings.

The ability of QUE and NAR to prevent or counteract seminal oxidative stress has been acknowledged on numerous occasions. Nevertheless, their affinity towards specific free radicals may differ, as revealed by our luminometric and colorimetric analyses. The NBT test, an assay designed to quantify the intracellular production of the superoxide radical [24], has shown that QUE was highly effective in suppressing the high levels of superoxide, which is considered to be the predominant ROS produced by biological systems and the first one to initiate the Fenton and Haber-Weiss reaction. Reports on the effects of QUE on human [41], rat [42], and bull spermatozoa [14] suggest that QUE could exhibit its anti-superoxide effects via the inhibition of NADPH oxidase and/or NADH-dependent oxidoreductase; superoxide dismutase mimicking; and a direct superoxide quenching. As such, we may speculate that QUE may be particularly useful during the first stages of possible oxidative chain reactions, keeping the superoxide production under control.

The luminometric assay enabling luminol as the probe serves to estimate the global concentration of ROS produced intra- as well as extracellularly. In this case, lower ROS amounts were detected in the case of NAR. As discussed previously, a number of studies have revealed a strong affinity of NAR towards H_2_O_2_, which has been reported to be produced in higher amounts than superoxide [43]. High H_2_O_2_ concentrations coupled with its membrane permeability and affinity towards the Fenton reaction, contribute to a large extent to oxidative damage of the sperm structures. This hypothesis is consistent with Sahin et al. [36], who observed a significant improvement of testicular cells of H_2_O_2_-treated rats following NAR treatment. Besides its ability to prevent H_2_O_2_ overproduction and to neutralize its effects, NAR has been shown to effectively neutralize hydroxyl radicals, nitric oxide, DPPH, and hypochlorous acid [44,45].

Protein carbonyl content is the most common indicator of protein oxidation. Carbonyl derivatives of proteins may occur as a product from oxidative modification of amino acid chains and reactive oxygen-mediated peptide cleavage [25]. In this study, QUE was able to decrease the extent of protein oxidation in stored boar spermatozoa. This effect may be directly related to the ROS-quenching potential of QUE, which may prevent any remaining ROS to possibly interact with the structure and/or function of proteins present in male reproductive cells. Nevertheless, previous data concerning the effects of natural biomolecules on sperm proteins are sparse and primarily focus on peptides playing an important role in the intracellular antioxidant defense mechanisms, such as reduced glutathione (GSH). Our data on the ability of particularly QUE to prevent oxidative damage of proteins are in agreement with Ben Abdallah et al. [46] and Bu et al. [47], who observed that QUE supplementation significantly restored the levels of depleted GSH and protected the activities of intracellular enzymes in testicular germ cells. Nevertheless, further studies on the specific mechanism of action by which natural antioxidants protect either structural or functional proteins from oxidative insults are most needed.

On the other hand, NAR exhibited a significant ability to prevent peroxidative damage to the sperm lipids. This property may be directly associated with the polar nature of NAR, which may facilitate its adherence to the lipid bilayer, and thus protect the sperm cell membrane [44]. Similarly to our findings, earlier studies have reported a significant decrease of LPO in male reproductive tissues and cells following NAR supplementation [18,48,49]. A similar hypothesis may be applied in the case of QUE, which has been partially confirmed in bovine and human spermatozoa [18,38,42]. What is more, Boots et al. [50] speculated that QUE was able to suppress LPO by inhibiting superoxide overproduction, by chelating transition metals crucial for the formation of hydroxyl radicals, and by suppressing the occurrence of lipoperoxides.

Summarizing our data, we may hypothesize that although both biomolecules exhibited beneficial effects on the sperm structural integrity and functional activity, the exact mechanism of action is different and specific to each flavonoid. NAR acts as a potent global ROS quencher, resulting in a decreased LPO and a subsequent plasma membrane, acrosome, and DNA stabilization, which has a direct impact on sperm motility. QUE on the other hand, exhibits strong mitochondrial-stabilizing effects, leading to the preservation of the mitochondrial activity and protein molecules, with a subsequent prevention of ROS overproduction, followed by the stabilization of sperm motility over a prolonged period of semen storage.

## 5. Conclusions

This study allowed a more complex definition of the in vitro impact of selected flavonoids on stored boar spermatozoa, which was time- and dose-dependent. The assessment of our data revealed that exposure of male gametes, particularly to 10 μM QUE and a concentration range of 10–25 μM NAR led to the preservation of sperm motility. While the lowest proportion of cells exhibiting disruptions of the membrane and acrosome integrity was detected in the experimental groups exposed to NAR, the highest mitochondrial activity was recorded in the case of QUE. Both biomolecules exhibited significant antioxidant properties, with QUE acting as a potent superoxide quencher, while NAR was able to decrease the global ROS concentration in a more effective way. On the one hand, QUE acted as a stronger protective agent against protein oxidation, while on the other hand, NAR was revealed to be more effective against lipid peroxidation.

Our results indicate that both biomolecules have the ability to interact with the structure and function of ejaculated spermatozoa during short-term storage. Nevertheless, further in vitro and in vivo fertilization assays following exposure to quercetin and naringenin are necessary. As such, the results of this study may serve as an important foundation for more complex assessments of the potential of flavonoids as alternative supplements to semen extenders.

## Figures and Tables

**Table 1:** Boar sperm motility (%) following exposure to quercetin or naringenin (*n* = 28).

	0 h	24 h	48 h	72 h
Ctrl	75.00 ± 7.33	52.25 ± 8.13	49.25 ± 6.94	46.00 ± 2.30
5 µM/L QUE	87.00 ± 3.74	83.00 ± 3.45 ***	69.25 ± 0.85 **	56.75 ± 2.74
10 µM/L QUE	84.50 ± 3.42	90.25 ± 3.33 ****	68.75 ± 8.15 **	57.00 ± 2.30 *
25 µM/L QUE	85.22 ± 3.80	77.75 ± 2.00 **	48.50 ± 1.90	26.75 ± 4.96 *
50 µM/L QUE	67.00 ± 6.14	38.75 ± 4.15	25.75 ± 1.39 *	2.00 ± 0.77 ****
5 µM/L NAR	78.50 ± 6.21	62.25 ± 3.96	60.25 ± 7.31	52.75 ± 4.64
10 µM/L NAR	79.25 ± 8.07	79.50 ± 3.00 ***	68.00 ± 4.06 **	54.25 ± 2.97
25 µM/L NAR	83.75 ± 5.72	69.75 ± 3.83 *	65.75 ± 5.43 *	59.00 ± 2.16 *
50 µM/L NAR	80.75 ± 6.41	69.00 ± 5.00 *	44.50 ± 8.07	35.00 ± 5.66

Mean ± S.D. * *p* < 0.05; ** *p* < 0.01; *** *p* < 0.001; **** *p* < 0.0001 in comparison with the control. QUE—quercetin; NAR—naringenin.

**Table 2:** Membrane integrity (%) of extended boar spermatozoa following exposure to quercetin or naringenin (*n* = 28).

	0 h	24 h	48 h	72 h
Ctrl	90.33 ± 2.84	79.67 ± 2.60	67.33 ± 1.25	50.67 ± 2.93
5 µM/L QUE	90.46 ± 4.25	86.33 ± 1.20	76.65 ± 1.56	66.56 ± 1.56 **
10 µM/L QUE	91.33 ± 4.09	89.67 ± 2.30 **	79.50 ± 1.00 **	72.67 ± 1.20 ***
25 µM/L QUE	91.07 ± 3.84	86.35 ± 1.74	71.68 ± 3.72	48.33 ± 4.41
50 µM/L QUE	93.00 ± 3.21	79.62 ± 0.57	54.33 ± 1.53 **	35.33 ± 2.89 **
5 µM/L NAR	91.65 ± 3.55	85.67 ± 1.76	77.00 ± 1.73	66.79 ± 4.05 ***
10 µM/L NAR	91.69 ± 3.66	89.37 ± 2.18 **	80.33 ± 2.84 **	78.00 ± 1.15 ****
25 µM/L NAR	91.54 ± 4.63	88.55 ± 0.89 *	77.33 ± 1.52 *	76.32 ± 2.18 ****
50 µM/L NAR	91.00 ± 2.08	84.67 ± 1.45	71.22 ± 3.93	48.00 ± 2.00

Mean ± S.D. * *p* < 0.05; ** *p* < 0.01; *** *p* < 0.001; **** *p* < 0.0001 in comparison with the control. QUE—quercetin; NAR—naringenin.

**Table 3:** Acrosome integrity (%) of extended boar spermatozoa following exposure to quercetin or naringenin (*n* = 28).

	0 h	24 h	48 h	72 h
Ctrl	93.00 ± 1.15	85.65 ± 2.75	68.95 ± 4.04	51.00 ± 4.26
5 µM/L QUE	93.54 ± 0.58	85.64 ± 3.82	74.68 ± 3.65	60.85 ± 3.21
10 µM/L QUE	92.00 ± 1.00	90.35 ± 3.25	79.67 ± 4.66 *	68.33 ± 3.06 **
25 µM/L QUE	92.55 ± 2.65	88.00 ± 2.64	73.45 ± 1.56	49.67 ± 3.56
50 µM/L QUE	92.63 ± 2.55	78.65 ± 3.25	50.00 ± 2.86 ***	32.36 ± 3.72 ***
5 µM/L NAR	94.53 ± 1.55	85.48 ± 3.09	72.69 ± 2.08	61.67 ± 4.51
10 µM/L NAR	93.67 ± 1.15	89.00 ± 1.98	79.00 ± 2.77 **	73.39 ± 3.52 ***
25 µM/L NAR	94.00 ± 1.00	90.59 ± 1.78	83.00 ± 2.65 **	71.55 ± 3.25 **
50 µM/L NAR	94.50 ± 2.54	84.00 ± 3.61	71.35 ± 3.54	52.33 ± 4.25

Mean ± S.D. * *p* < 0.05; ** *p* < 0.01; *** *p* < 0.001; **** *p* < 0.0001 in comparison with the Ccntrol. QUE—quercetin; NAR—naringenin.

**Table 4:** Boar sperm DNA fragmentation (%) following exposure to quercetin or naringenin (*n* = 28).

	0 h	24 h	48 h	72 h
Ctrl	5.48 ± 0.49	9.87 ± 0.77	12.53 ± 1.35	17.01 ± 1.00
5 µM/L QUE	4.96 ± 0.49	8.79 ± 0.68	10.58 ± 1.54	14.16 ± 2.25
10 µM/L QUE	5.18 ± 0.59	6.01 ± 1.00 *	6.73 ± 1.62 *	10.21 ± 1.51 *
25 µM/L QUE	5.51 ± 0.65	6.91 ± 0.53	8.95 ± 1.07	18.11 ± 3.81
50 µM/L QUE	4.99 ± 0.48	11.53 ± 0.88	17.67 ± 2.03 *	22.62 ± 2.93 *
5 µM/L NAR	5.01 ± 0.58	8.46 ± 0.73	10.64 ± 0.45	14.49 ± 3.29
10 µM/L NAR	5.06 ± 0.52	6.77 ± 0.67 *	7.96 ± 0.19 *	11.78 ± 1.34 *
25 µM/L NAR	5.07 ± 0.26	5.67 ± 1.20 *	6.79 ± 0.36 *	10.70 ± 0.62 *
50 µM/L NAR	5.82 ± 0.51	7.07 ± 0.93	15.42 ± 1.01	16.80 ± 2.29

Mean ± S.D. * *p* < 0.05 in comparison with the control. QUE—quercetin; NAR—naringenin.

**Table 5:** The effect of quercetin and naringenin on the reactive oxygen species (ROS) production (RLU/s/10^6^ sperm) by extended boar spermatozoa (*n* = 28).

	0 h	24 h	48 h	72 h
Ctrl	6.92 ± 0.93	15.00 ± 1.73	23.67 ± 2.98	33.53 ± 3.25
5 µM/L QUE	6.47 ± 0.91	10.33 ± 1.55	18.00 ± 1.75	25.64 ± 1.59 *
10 µM/L QUE	5.93 ± 0.70	9.36 ± 1.85 *	15.64 ± 2.03 **	21.38 ± 2.05 ***
25 µM/L QUE	6.73 ± 0.46	10.55 ± 1.47	22.64 ± 1.89	26.15 ± 1.15
50 µM/L QUE	7.32 ± 0.65	16.58 ± 2.05	29.58 ± 1.58	38.73 ± 2.08
5 µM/L NAR	6.41 ± 0.68	9.36 ± 1.74 *	16.54 ± 1.15 *	21.33 ± 3.05 ***
10 µM/L NAR	5.73 ± 0.82	8.35 ± 0.56 *	15.63 ± 0.59 **	19.25 ± 1.87 ****
25 µM/L NAR	6.03 ± 0.95	8.67 ± 0.89 *	12.85 ± 1.59 ***	18.33 ± 1.79 ****
50 µM/L NAR	6.76 ± 0.58	11.69 ± 1.76	20.00 ± 1.73	31.00 ± 3.61

Mean ± S.D. * *p* < 0.05; ** *p* < 0.01; *** *p* < 0.001; **** *p* < 0.0001 in comparison with the control. QUE—quercetin; NAR—naringenin.

**Table 6:** The effect of quercetin and naringenin on the amount of protein carbonyls (nmol/mg protein) in extended boar spermatozoa (*n* = 28).

	0 h	24 h	48 h	72 h
Ctrl	0.75 ± 0.15	1.65 ± 0.42	2.68 ± 0.59	5.09 ± 0.56
5 µM/L QUE	0.79 ± 0.19	1.45 ± 0.36	1.98 ± 0.15	3.67 ± 0.67 *
10 µM/L QUE	0.77 ± 0.13	1.27 ± 0.36	1.89 ± 0.21	2.92 ± 0.56 ***
25 µM/L QUE	0.73 ± 0.16	1.19 ± 0.35	2.63 ± 0.36	4.92 ± 0.45
50 µM/L QUE	0.72 ± 0.29	2.14 ± 0.25	2.75 ± 0.70	6.08 ± 0.43
5 µM/L NAR	0.82 ± 0.07	1.52 ± 0.28	2.45 ± 0.29	4.51 ± 0.45
10 µM/L NAR	0.76 ± 0.14	1.45 ± 0.38	2.32 ± 0.41	3.92 ± 0.55
25 µM/L NAR	0.78 ± 0.17	1.35 ± 0.23	2.15 ± 0.34	3.47 ± 0.49 *
50 µM/L NAR	0.83 ± 0.17	1.80 ± 0.49	2.66 ± 0.31	5.36 ± 0.52

Mean ± S.D. * *p* < 0.05; *** *p* < 0.001 in comparison with the control. QUE—quercetin; NAR—naringenin.

**Table 7:** The effect of quercetin and naringenin on the amount of malondialdehyde (μmol/g protein) in extended boar spermatozoa (*n* = 28).

	0 h	24 h	48 h	72 h
Ctrl	4.43 ± 0.71	9.09 ± 0.56	14.69 ± 0.43	20.42 ± 0.25
5 µM/L QUE	4.19 ± 0.65	8.76 ± 0.92	10.48 ± 0.42	18.34 ± 1.18
10 µM/L QUE	4.71 ± 0.30	8.33 ± 0.80	9.85 ± 1.03 *	15.42 ± 1.08 *
25 µM/L QUE	4.26 ± 0.70	7.62 ± 0.35	12.49 ± 1.05	20.69 ± 2.05
50 µM/L QUE	4.49 ± 0.45	10.16 ± 0.49	15.52 ± 1.57	24.19 ± 0.81
5 µM/L NAR	4.65 ± 0.49	8.40 ± 0.38	11.12 ± 0.95	20.11 ± 0.34
10 µM/L NAR	4.26 ± 0.70	7.59 ± 0.31	9.16 ± 0.71 *	14.10 ± 1.62 **
25 µM/L NAR	4.49 ± 0.54	6.85 ± 0.49 *	8.88 ± 0.82 *	13.78 ± 0.86 **
50 µM/L NAR	4.65 ± 0.49	9.33 ± 0.88	12.72 ± 2.62	21.67 ± 1.85

Mean ± S.D. * *p* < 0.05; ** *p* < 0.01 in comparison with the control. QUE—quercetin; NAR—naringenin.
